# Using human induced pluripotent stem cells to model cerebellar disease: Hope and hype

**DOI:** 10.3109/01677063.2015.1053478

**Published:** 2015-08-27

**Authors:** Sarah Wiethoff, Charles Arber, Abi Li, Selina Wray, Henry Houlden, Rickie Patani

**Affiliations:** ^a^ National Hospital for Neurology and Neurosurgery, UCL Institute of Neurology, London, UK; ^b^ Center for Neurology and Hertie Institute for Clinical Brain Research, Eberhard-Karls-University, Tübingen, Germany; ^c^ Department of Molecular Neuroscience and Queen Square Brain Bank, UCL Institute of Neurology, London, UK; ^d^ Department of Clinical Neurosciences, University of Cambridge, Cambridge, UK; ^e^ Euan MacDonald Centre for MND, University of Edinburgh, Edinburgh, UK

**Keywords:** Cerebellum, directed differentiation, disease modelling, iPSC-technology, reprogramming

## Abstract

The cerebellum forms a highly ordered and indispensible component of motor function within the adult neuraxis, consisting of several distinct cellular subtypes. Cerebellar disease, through a variety of genetic and acquired causes, results in the loss of function of defined subclasses of neurons, and remains a significant and untreatable health care burden. The scarcity of therapies in this arena can partially be explained by unresolved disease mechanisms due to inaccessibility of human cerebellar neurons in a relevant experimental context where initiating disease mechanisms could be functionally elucidated, or drug screens conducted. In this review we discuss the potential promise of human induced pluripotent stem cells (hiPSCs) for regenerative neurology, with a particular emphasis on in vitro modelling of cerebellar degeneration. We discuss progress made thus far using hiPSC-based models of neurodegeneration, noting the relatively slower pace of discovery made in modelling cerebellar dysfunction. We conclude by speculating how strategies attempting cerebellar differentiation from hiPSCs can be refined to allow the generation of accurate disease models. This in turn will permit a greater understanding of cerebellar pathophysiology to inform mechanistically rationalised therapies, which are desperately needed in this field.

## Introduction

The cerebellum, also termed ‘little brain’, is an extensively researched part of the neuraxis. Nonetheless, the molecular pathogenesis underlying cerebellar disease remains poorly understood, with the vast majority of such disorders still being immedicable to date (Manto, 2008). As one of the more principal neuraxial structures with high cellular and structural complexity, the cerebellum integrates and orchestrates ∼80% of the number of total CNS neurons in only 10% of the total volume (Azevedo et al., 2009). Its anatomical location in the posterior cranial fossa is functionally related to coordinating the major input from pontine and vestibular nuclei, the inferior olive and spinal cord to the cerebellum's major output pathways via the ventrolateral thalamus and pontine, medullary reticular, red and vestibular nuclei (De Zeeuw & Berrebi, 1995; Ito, 1984; Voogd et al., 1996; Ruigrok, 2011). Using non-induced pluripotent stem cell (iPSC)-based approaches, the cerebellum has been extensively studied and has well-established functions in motor control and motor learning, (fine) coordination, posture and balance (Ito, 1984; Glickstein et al., 2009; Schmahmann, 1997). Additionally, there is growing evidence implicating the cerebellum in cognitive functions and emotion (Strata, 2015; Strick et al., 2009; Anand et al., 1959; Timmann, 2012). Its unique organisation, precise wiring and distinct pathophysiology make this particular structure equally vulnerable to injury and difficult to repair.

Regenerative medicine has been revolutionised by the potential of disease modelling and manipulation via hiPSC technology, a recent discovery later recognised by the Nobel Prize in Physiology or Medicine (Takahashi et al., 2007; Tabar & Studer, 2014). Differentiation of hiPSCs from healthy and diseased individuals into neuronal cells permits investigation and modelling of human neurodevelopmental processes. It also significantly fosters our understanding of the pathological processes driving different developmental and degenerative diseases and thus informs strategies to ameliorate disease progression, with the ultimate aim to restore structure and function (Xie & Zhang, 2015). Specific advantages include not only directed differentiation of any human cell type to model disease in a highly reductionist fashion but also that these cells will express mutations at a normal pathophysiological level, removing the need for artificial overexpression, knockdown or knockout. Furthermore, given their theoretically limitless potential to proliferate before directed differentiation into clinically relevant cell types, this ‘renewable’ resource for experimental material both recapitulates disease phenotypes and further enables high-throughput drug screening in search of a revertive therapeutic agent (Efthymiou et al., 2014; Malik et al., 2014; Yang et al., 2013). A second more long-term promise of hiPSCs will be patient-targeted individual therapy through transplantation, although accurate restoration of circuit integrity and function after damage will be a formidable task.

The advent of hiPSCs is of particular relevance to a structure like the cerebellum both from developmental and clinical neurological perspectives as most of the mysteries remain to be unravelled (Manto, 2008; Leiner, 2010; Voogd, 2014) and no cure is available for the treatment of the vast majority of cerebellar diseases. Against this background, understanding the developmental ‘logic’ of cerebellar differentiation is essential to first permit reproducible directed differentiation of hiPSCs in vitro, in order to generate accurate disease models for a variety of cerebellar disorders.

Here we highlight the promise of iPSC technology to regenerative medicine, and especially neurology with a specific focus on the cerebellum. We first discuss the prospects and remaining challenges of iPSC technology before focussing specifically on current strategies, achievements and limitations for directed differentiation to cerebellar neurons. We conclude by summarising the advances in modelling human cerebellar degeneration using hiPSCs and speculating on future areas of interest at the experimental interface between cerebellar neurology and human stem cell-based disease modelling ‘in-a-dish’.

## iPSC technology and its relevance to medicine

**Figure 1.**
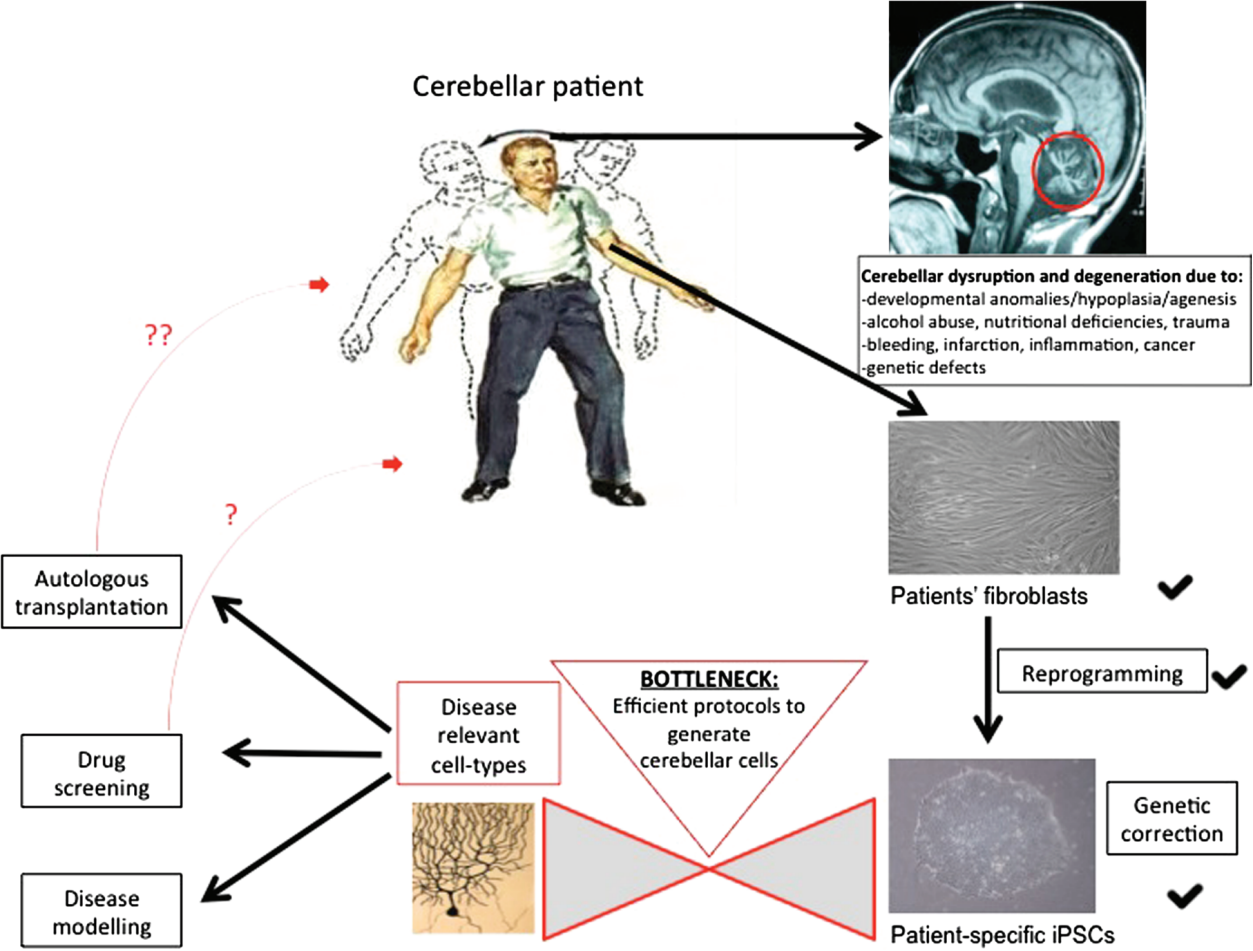
iPSC technology has already started to foster the study of the human cerebellum and its pathologies in a patient-specific way. Overcoming the current bottleneck of directed differentiation will further facilitate the beneficial effects of this technology on the disorders of the cerebellum. *Cerebellar Patient taken from Netter's Concise Neurology, p.85, Elsevier, Inc. (copyright).*

The discovery of efficient reprogramming of adult human fibroblasts into iPSCs in 2007 (Takahashi et al., 2007) has since caused a fundamental paradigm shift in regenerative medicine. Subsequent technique optimisation and refinement of reproducibility and scalability (Okita et al., 2011; Schlaeger et al., 2015; Yu et al., 2009; Jiang et al., 2013; Hou et al., 2013; Warren et al., 2010) have only reinforced the importance of hiPSC-based modelling. With the help of a small number of transgenes it is becoming a standardised procedure to revert primary somatic cells of diseased patients and healthy control individuals into iPSCs for many laboratories worldwide, indeed this technology has also attracted considerable (and growing) commercial interest. These reprogramming methods somewhat circumvent ethical concerns accompanied by the use of primary human embryonic stem cells (hESCs) for medical research, and revolutionise predating approaches including somatic nuclear transfer into oocytes or fusion with ESCs, leading to tetraploidy (Yamanaka & Blau, 2010). The theoretically endless capability of self-renewal and the preserved genetic background (where mutated genes are expressed at a representative pathophysiological level) render hiPSCs an unprecedented and unparalleled resource. Combined with ongoing advances in the field of directed differentiation into disease-specific cell types (Tabar & Studer, 2014; Shi et al., 2012; Schwartz et al., 2014; Teng et al., 2014; Dimos et al., 2008; Moretti et al., 2010), hiPSC in vitro models faithfully recapitulate key aspects not only of human development, but also of genetic and sporadic disease that would otherwise be inaccessible for investigation. Thus, and for the first time in regenerative medicine, this technology enables careful temporal interrogation of pathogenic events in a patient-specific manner using a fully humanised model, which bypasses the need for artificial overexpression, knockdown or knockout (for a schematic, see Figure 1).

## iPSC technology and its relevance to neurology

Neuronal cells have been amongst the earliest cell types to be generated via reproducible and efficient differentiation protocols from hESCs (Reubinoff et al., 2001; Zhang et al., 2001) and hiPSCs (Shi et al., 2012; Chambers et al., 2009). Protocols range from in vitro formation of embryoid bodies exposed to retinoic acid in a stage-specific manner, co-culture with neural inducing feeder cells or direct pharmacological inhibition of transforming growth factor (TGF-beta) and bone morphogenetic protein (BMP) signalling performed on a monolayer of stem cells (dual SMAD inhibition). These approaches can reliably induce neural conversion; the resulting precursors can be ‘patterned’ using developmentally rationalised morphogenetic cues, and then be terminally differentiated into region-specific neuronal and glial subtypes.

Although this technology permits modelling of human developmental processes and diseases (Lancaster et al., 2013; Marchetto et al., 2010) in an essentially foetal system (Patani et al., 2012), an ever growing number of studies confirm that mutant hiPSCs derived from patients with inherited disease can successfully recapitulate key cellular pathophysiology, including those diseases that are adult onset (Dimos et al., 2008; Israel et al., 2012; Koch et al., 2011; Lee et al., 2009; Sanchez-Danes et al., 2012; Schondorf et al., 2014; Woodard et al., 2014). In addition to uncovering cellular and molecular pathogenesis in inherited and sporadic conditions (Israel et al., 2012; Sanchez-Danes et al., 2012) hiPSCs can also be used successfully to screen for potential novel therapeutics (Yang et al., 2013; Bellin et al., 2012; Cooper et al., 2012) or to delineate genetic from non-genetic influences driving neuronal disease as demonstrated lately for the case of monogenic twins discordant in their clinical phenotype with Parkinson's disease (PD) (Woodard et al., 2014). While a comprehensive summary of the most important findings from iPSC studies in the field of neurology is beyond the scope of this article, the interested reader is referred to recent reviews on this topic (Xie & Zhang, 2015; Cao et al., 2014; Ross & Akimov, 2014). Despite this undisputed contribution to understanding development and disease of the human nervous system, remaining caveats include possible oncogene reactivation via reprogramming procedures, uncontrolled epigenetic and genetic modifications of generated stem cells and precursors from in vitro culture conditions or undiscovered transgene integration, low-efficiency and non-directed differentiation that might lead to proliferation and teratoma formation in vivo, laborious and expensive culture and experimental settings with high intrinsic biological variability. These will require further optimisation until this technique can realise its full potential for disease modelling, drug discovery and ultimately autologous transplantation, although this remains a much longer term aim with several unresolved issues to date (Tabar & Studer, 2014; Cyranoski, 2013; Grskovic et al., 2011; Ausubel et al., 2011; Frantz, 2012; Schwartz et al., 2012; Kordower et al., 2008).

### What distinguishes cerebellar modelling with iPSCs from the successful modelling of other subregions of the human brain?

Modelling cerebellar disease with hiPSC technology has not yet become a broadly used routine approach and is reported with less frequency compared with other neurological diseases.

Progress in modelling cortical, striatal, midbrain, spinal, peripheral sensory and autonomic nervous system development and disease using human pluripotent stem cells is largely attributable to the presence of established protocols for directed differentiation to region specific neurons and glia (Shi et al., 2012; Israel et al., 2012; Aubry et al., 2008; Delli Carri et al., 2013; Perrier & Peschanski, 2012; Jeon et al., 2012; Sanchez-Danes et al., 2012; Perrier et al., 2004; Yan et al., 2005; Kriks et al., 2011; Li et al., 2005, 2008; Hu & Zhang, 2009; Patani et al., 2011; Corti et al., 2012; Devlin et al., 2015; Valensi-Kurtz et al., 2010; Chambers et al., 2012; Lee et al., 2012; Pomp et al., 2005). This clearly fosters the applicability of hiPSCs in clinical research and further paves its way towards translation as reflected in the number of publications generated in these respective fields. Conversely, modelling cerebellar conditions has been hampered by the absence of such robust differentiation paradigms. Comparing the number of iPSC publications investigating neurological non-cerebellar disorders to the amount of published work harnessing iPSC technology to study cerebellar pathology we currently observe a strong disequilibrium: (1) the few reports studying cerebellar diseases with iPSC technology (Koch et al., 2011; Eigentler et al., 2013; Hick et al., 2013; Bird et al., 2014) have derived non-cerebellar cells from patients with primarily cerebellar phenotypes, (2) no reported study thus far has reproduced the single published protocol to specify cerebellar-like cells from hiPSCs (Erceg et al., 2012) available at that time.

### Why is the cerebellum lagging behind?

An important contribution to the lack of reliable and efficient cerebellar differentiation protocols might certainly come from the rarity of cerebellar diseases. One could argue that simply the medical and socio-economic necessity to generate hiPSC models of cerebellar disorders is lower and therefore orchestrated efforts of developmental biologists have focussed on deriving more ‘deserving’ cell types for more common disorders such as PD and Alzheimer's disease (AD). In these disciplines, significant progress in directed differentiation of disease-relevant neurons has been made (Shi et al., 2012; Perrier et al., 2004; Yan et al., 2005; Kriks et al., 2011). Even though (hereditary) degenerative conditions of the cerebellum might be less frequent than degenerative conditions of the basal ganglia or cortex, the cerebellum is affected in a range of common and rare conditions, including paraneoplastic cerebellar degeneration, alcohol abuse, nutritional deficiency and rare inherited conditions [dominant and recessive spinocerebellar ataxias (SCAs and ARCAs)]. Moreover, multiple sclerosis – the most common cause for neurological disability in young people of developed countries – frequently targets the cerebellum, and tumours in the granule cell (medulloblastomas) represent up to 20–40% of brain tumours in children and young adults (Arseni & Ciurea, 1981; Salero & Hatten, 2007). Additionally, cerebellar malformations (e.g. Dandy–Walker or Arnold–Chiari malformations, Joubert syndrome and pontocerebellar hypoplasias) are highly disabling both to the affected patients and their carers. Currently, we cannot offer treatment to prevent ongoing cerebellar degeneration, and we will not be able to in the near future, unless we understand the molecular and cellular mechanisms underlying human cerebellar degeneration more precisely. The generation of reliable disease models will be crucial to progress in this field. We therefore want to highlight that a deeper mechanistic understanding of cerebellar development will permit establishment of accurate disease models using iPSC technology. Insights into the molecular pathogenesis will in turn allow more mechanistically rationalised approaches to therapy in this arena.

Against this background, it seems plausible that the inherent and notorious developmental complexity of cerebellar cells [and especially Purkinje cells (PCs)] and subsequent difficulties generating them from human iPSCs have hampered significant progress in this field, despite both early (Salero & Hatten, 2007; Su et al., 2006; Muguruma et al., 2010) and indeed more recent developmental breakthroughs (Muguruma et al., 2015).

### What has been done to study cerebellar diseases using iPSC technology?

At the time of writing, the authors are aware of one publication using hiPSC-derived long-term self-renewing neuroepithelial-like stem (lt-NES) cells from patients with SCA3 to study the cellular pathology of the most common autosomal-dominant degenerative cerebellar disorder (Koch et al., 2011). Here, the inherent difficulty and the lack of efficient in vitro cerebellar differentiation protocols are circumvented by studying lt-NES cells. These cells do express a hindbrain-like transcriptional signature with markers usually observed in cells of a ventral anterior hindbrain fate (Koch et al., 2009) which transcriptionally (and possibly functionally) approximates these cells to the cerebellum. Using this model, the authors detected neuron-specific early aberrant protein processing, aggravated by excitation in SCA3 mutant iPSC neurons, thereby breaking a milestone for the field in modelling late-onset cerebellar disease in a dish (Koch et al., 2011). Since then, Friedreich's ataxia, the most common autosomal recessive cerebellar disorder with additional multisystemic affection has been studied through various attempts using iPSC technology. Again, investigators in these studies utilised non-cerebellar cells to study a cellular phenotype (Eigentler et al., 2013; Hick et al., 2013; Bird et al., 2014; Ku et al., 2010). None of the above studies reported successful utilisation or modification of the – by that time – only published protocol describing cerebellar-like granule cell differentiation from hiPSCs (Erceg et al., 2012).

In order to explain the lack of studies using patient-derived iPSCs to generate cerebellar cells and study their pathology we need to address the following question:

### What do we know about the development of erebellar cells that might distinguish them from other neuronal cells?

Cerebellar development in humans occurs over a prolonged time span ranging from the early embryonic period to the first postnatal years. It happens in parallel to the development of the forebrain, midbrain, spinal cord and their arising substructures, but follows its own distinct stereotyped pattern. In summary, four basic processes can be delineated: Firstly, the cerebellar primordium forms at the midbrain–hindbrain boundary (MHB) under close transcriptional influence of the isthmic organiser (IsO; for more information on this structure, see, Wurst & Bally-Cuif, 2001). Subsequently, two different proliferative compartments appear, giving rise to two distinct principal cerebellar cell type precursors, which will later migrate and/or differentiate to form the inhibitory PCs and the excitatory granule cells. In a third step, the aforementioned granule precursors migrate tangentially to form the external granule layer (EGL) where significant maturation takes place before they eventually migrate radially inwards to their ultimate residence in the internal granule layer (IGL). Finally, cell maturation and establishment of cellular connections characterise the last process giving rise to the three-layered cerebellar cortex with its distinct circuitry and conserved foliation pattern that organises the cerebellum into 10 lobules (for more detailed information on cerebellar development, please see, Ten Donkelaar & Lammens, 2009; White & Sillitoe, 2013). It is noteworthy that the cerebellar cortex is connected to the remaining neuraxis only via long projecting axons arising from PC somata in the central PC layer, which exert modulatory inhibitory input to deep cerebellar nuclei (DCN).

### What do we know about the development of Purkinje cells that might distinguish them from other cerebellar cells?

PCs are not only the cerebellum's key effectors but they are also thought to be primarily affected in SCAs (Hekman & Gomez, 2015) among other cerebellar conditions. Their pronounced sensitivity, morphology, and unique functional circuitry with neighbouring cells permits a carefully coordinated output, which integrates complex temporal and spatial events at the synapse to long-term depression (LTD), or long-term potentiation (LTP) with precision and fidelity (White & Sillitoe, 2013; Schorge et al., 2010; Buffo & Rossi, 2013).

Their embryonic origin stems from neural progenitor cells of the cerebellar ventricular zone (Hoshino, 2006; Hibi & Shimizu, 2012), one of the two germinal layers of the cerebellar primordium. However, it has been intrinsically difficult for developmental biologists to influence PC generation either in vivo or in vitro. Although publication bias towards positive findings confounds an accurate and comprehensive analysis of past and current attempts at cerebellar neurogenesis, one notable recent study demonstrates successful transplantation of cerebellar neural stem cells (NSC) into the cerebellum of SCA3 mutant mice with functional reorganisation (Mendonca et al., 2015). This report confirmed a reduction of mutant ataxin-3 (ATXN3) inclusions and atrophy within the cellular layers of the cerebellum and specific preservation of the number of PCs post-transplant compared with the non-transplanted mutant mice. Importantly this work failed to demonstrate the specification of PCs from the cerebellar NSC graft in vivo and in vitro, thus suggesting an indirect beneficial effect of the graft on PC survival and cerebellar integrity. The differentiation of TUJ1-positive neurons, glial fibrillary acidic protein (GFAP)-positive astrocytes and neural/glial antigen 2 (NG2)-positive oligodendrocytes and their subsequent integration into the cerebellum was observed in vivo and in vitro in their mouse model, at the expense of PCs. These results seem to be somewhat mirrored by the advances in the field of cerebellar cell differentiation from hiPSCs: whilst there has been no successful report of mature fully functional PC differentiation from hiPSCs or ESCs until early 2015 (Muguruma et al., 2015; Wang et al., 2015), generation of MATH1 + cerebellar-like granule cells from hiPSCs had been reported years earlier (Erceg et al., 2012).

### How can the field move from here?

The recent publication of Muguruma et al. (2015) is a landmark study recapitulating key developmental steps in cerebellar neurogenesis in order to achieve efficient directed differentiation of hiPSCs. Briefly, the authors report regionalising ESC-derived embryoid bodies to the MHB in vitro and subsequent generation of self-organising cerebellar plate neuroepithelium (CPNE) that gives rise to mature, fully functional PCs and granule cells, as well DCN neurons and various interneurons in specific co-culture settings. By the precise timing of sequentially administered extrinsic morphogenetic signals [fibroblast growth factor2 (FGF2), FGF19 and stromal cell-derived factor 1 (SDF1)] the team promoted self-formation of continuous CPNE with dorsal-ventral and apical-basal organisation, mimicking cerebellar development for the first time in a dish. The investigators managed to harness the currently pitted knowledge of developmental cues required for cerebellar development to a reductionist 3D culture with external administration of three morphogens in a timely defined manner only. This proof of principle of cerebellar-like development in vitro was conducted on hESCs, although the authors also confirm that the same protocol applied to two hiPSC lines yielded similar findings (Muguruma et al., 2015). This study will certainly inspire researchers to reproduce, modify (Wang et al., 2015) and further optimise the protocol (e.g. monolayer culture in fully chemically defined medium) and to use this platform to further investigate human cerebellar development and degeneration (see Figure 1).

## The future of iPSC technology in modelling cerebellar diseases

**Figure 2.**
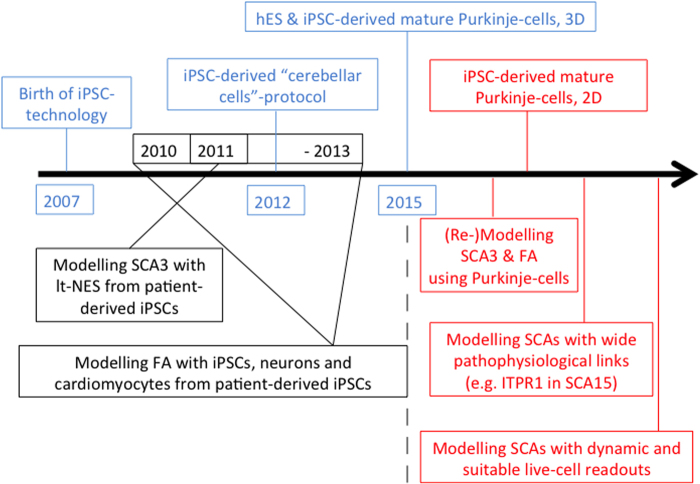
Modelling cerebellar diseases with hiPSC technology: past, present and future. We think, SCA3 and Friedreich's ataxia (FA), as the most common autosomal-dominant and -recessive cerebellar degenerative conditions, should be remodelled using PCs differentiated from patient-derived iPSCs once possible. Furthermore, preferential choice should target cerebellar diseases due to genetic defects in pathophysiologically widely linked genes, e.g. SCA15 (Schorge et al., 2010), and employ suitable cellular readouts with a focus on electrophysiology and live-cell imaging to widen our knowledge about cerebellar diseases as ‘impaired network and impaired plasticity’ disorders.

If the above-mentioned goal(s) can be met, the future of modelling cerebellar diseases with iPSC technology will be bright. We are cautiously optimistic that efficient and reproducible protocols for the generation of PCs and granule cells from hiPSCs will be developed within the coming years using insights from developmental biology and also guided by recent breakthroughs (Koch et al., 2011; Muguruma et al., 2015; Mendonca et al., 2015). This will be crucially important to foster this currently underrepresented field within hiPSC modelling of neurodegeneration. Such advances will permit accurate study of developmental and degenerative processes. Here, having lagged behind the main field previously might actually become a strategic advantage: important platforms for disease modelling (for recent reviews, please see e.g., Zeng et al., 2014), drug discovery (e.g., Hunsberger et al., 2015) and evaluation of cell replacement therapies (Kamao et al., 2014) have been established in the meantime successfully for other iPSC-derived cell types, and can be adapted to iPSC-derived cerebellar cells by building on this important knowledge. Successfully applied, these platforms will exert their full potential towards a better understanding of cerebellar development, disease pathways and therapeutic avenues in cerebellar disease. We depict this in a time bar summarising past efforts (blue and black font) with future predictions (red font) which may be of special interest to model genetic cerebellar diseases with iPSC technology (Figure 2). Furthermore, efforts in establishing accurate cerebellar in vitro disease models will – as has happened in other fields already (Soldner et al., 2011) – be complemented by the generation of isogenic hiPSC lines via modern genomic editing techniques (Hockemeyer et al., 2009, 2011). Such approaches allow introducing or ‘correcting’ different mutations that solely affect cerebellar integrity against an identical genetic background to precisely disentangle disease-causing mechanisms from background genomic variation between different lines. Finally, the gathered knowledge and progress towards efficient cerebellar differentiation protocols will impact on direct lineage reprogramming efforts (for recent reviews, please see, Ladewig et al., 2013; Vierbuchen & Wernig, 2012).

In summary, the cerebellum currently fails to obtain comparable experimental recognition as other neuraxial regions largely due to a poor translation of its underlying developmental biology into directed differentiation strategies. Noting recent breakthroughs in the directed differentiation of human pluripotent stem cells into cerebellar derivatives, we predict that this hitherto relatively understudied region of the neuraxis can now begin to receive the experimental attention it deserves. This in turn may illuminate the precise mechanisms of cerebellar development and degeneration, thus informing therapeutic strategy.

## References

[CIT0001] Anand B. K., Malhotra C. L., Singh B, Dua S ((1959)). Cerebellar projections to limbic system. J Neurophysiol.

[CIT0002] Arseni C, Ciurea A. V. ((1981)). Statistical survey of 276 cases of medulloblastoma (1935–1978). Acta Neurochir.

[CIT0003] Aubry L, Bugi A, Lefort N, Rousseau F, Peschanski M, Perrier A. L. ((2008)). Striatal progenitors derived from human ES cells mature into DARPP32 neurons in vitro and in quinolinic acid-lesioned rats. Proc Natl Acad Sci U S A.

[CIT0004] Ausubel L. J., Lopez P. M., Couture L. A. ((2011)). GMP scale-up and banking of pluripotent stem cells for cellular therapy applications. Methods Mol Biol.

[CIT0005] Azevedo F. A., Carvalho L. R., Grinberg L. T., Farfel J. M., Ferretti R. E., Leite R. E. ((2009)). Equal numbers of neuronal and nonneuronal cells make the human brain an isometrically scaled-up primate brain. J Comp Neurol.

[CIT0006] Bellin M, Marchetto M. C., Gage F. H., Mummery C. L. ((2012)). Induced pluripotent stem cells: The new patient?. Nat Rev Mol Cell Biol.

[CIT0007] Bird M. J., Needham K, Frazier A. E., van Rooijen J, Leung J, Hough S ((2014)). Functional characterization of Friedreich ataxia iPS-derived neuronal progenitors and their integration in the adult brain. PLoS one.

[CIT0008] Buffo A, Rossi F ((2013)). Origin, lineage and function of cerebellar glia. Prog Neurobiol.

[CIT0009] Cao L, Tan L, Jiang T, Zhu X. C., Yu J. T. ((2014)). Induced pluripotent stem cells for disease modeling and drug discovery in neurodegenerative diseases. Mol Neurobiol.

[CIT0010] Chambers S. M., Fasano C. A., Papapetrou E. P., Tomishima M, Sadelain M, Studer L ((2009)). Highly efficient neural conversion of human ES and iPS cells by dual inhibition of SMAD signaling. Nat Biotechnol.

[CIT0011] Chambers S. M., Qi Y, Mica Y, Lee G, Zhang X. J., Niu L ((2012)). Combined small-molecule inhibition accelerates developmental timing and converts human pluripotent stem cells into nociceptors. Nat Biotechnol.

[CIT0012] Cooper O, Seo H, Andrabi S, Guardia-Laguarta C, Graziotto J, Sundberg M ((2012)). Pharmacological rescue of mitochondrial deficits in iPSC-derived neural cells from patients with familial Parkinson's disease. Sci Transl Med.

[CIT0013] Corti S, Nizzardo M, Simone C, Falcone M, Nardini M, Ronchi D ((2012)). Genetic correction of human induced pluripotent stem cells from patients with spinal muscular atrophy. Sci Transl Med.

[CIT0014] Cyranoski D ((2013)). Stem cells cruise to clinic. Nature.

[CIT0015] De Zeeuw C. I., Berrebi A. S. ((1995)). Postsynaptic targets of Purkinje cell terminals in the cerebellar and vestibular nuclei of the rat. Eur J Neurosci.

[CIT0016] Delli Carri A, Onorati M, Lelos M. J., Castiglioni V, Faedo A, Menon R ((2013)). Developmentally coordinated extrinsic signals drive human pluripotent stem cell differentiation toward authentic DARPP-32 + medium-sized spiny neurons. Development.

[CIT0017] Devlin A. C., Burr K, Borooah S, Foster J. D., Cleary E. M., Geti I ((2015)). Human iPSC-derived motoneurons harbouring TARDBP or C9ORF72 ALS mutations are dysfunctional despite maintaining viability. Nat commun.

[CIT0018] Dimos J. T., Rodolfa K. T., Niakan K. K., Weisenthal L. M., Mitsumoto H, Chung W ((2008)). Induced pluripotent stem cells generated from patients with ALS can be differentiated into motor neurons. Science.

[CIT0019] Efthymiou A, Shaltouki A, Steiner J. P., Jha B, Heman-Ackah S. M., Swistowski A ((2014)). Functional screening assays with neurons generated from pluripotent stem cell-derived neural stem cells. J Biomol Screen.

[CIT0020] Eigentler A, Boesch S, Schneider R, Dechant G, Nat R ((2013)). Induced pluripotent stem cells from friedreich ataxia patients fail to upregulate frataxin during in vitro differentiation to peripheral sensory neurons. Stem Cells Dev.

[CIT0021] Erceg S, Lukovic D, Moreno-Manzano V, Stojkovic M, Bhattacharya S. S. ((2012)). Derivation of cerebellar neurons from human pluripotent stem cells. Curr Protoc Stem Cell Biol, Chapter 1.

[CIT0022] Frantz S ((2012)). Embryonic stem cell pioneer Geron exits field, cuts losses. Nat Biotechnol.

[CIT0023] Glickstein M, Strata P, Voogd J ((2009)). Cerebellum: History. Neuroscience.

[CIT0024] Grskovic M, Javaherian A, Strulovici B, Daley G. Q. ((2011)). Induced pluripotent stem cells–opportunities for disease modelling and drug discovery. Nat Rev Drug Discov.

[CIT0025] Hekman K. E., Gomez C. M. ((2015)). The autosomal dominant spinocerebellar ataxias: Emerging mechanistic themes suggest pervasive Purkinje cell vulnerability. J Neurol Neurosurg Psychiatry.

[CIT0026] Hibi M, Shimizu T ((2012)). Development of the cerebellum and cerebellar neural circuits. Dev Neurobiol.

[CIT0027] Hick A, Wattenhofer-Donze M, Chintawar S, Tropel P, Simard J. P., Vaucamps N ((2013)). Neurons and cardiomyocytes derived from induced pluripotent stem cells as a model for mitochondrial defects in Friedreich's ataxia. Dis Model Mech.

[CIT0028] Hockemeyer D, Soldner F, Beard C, Gao Q, Mitalipova M, DeKelver R. C. ((2009)). Efficient targeting of expressed and silent genes in human ESCs and iPSCs using zinc-finger nucleases. Nat Biotechnol.

[CIT0029] Hockemeyer D, Wang H, Kiani S, Lai C. S., Gao Q, Cassady J. P. ((2011)). Genetic engineering of human pluripotent cells using TALE nucleases. Nat Biotechnol.

[CIT0030] Hoshino M ((2006)). Molecular machinery governing GABAergic neuron specification in the cerebellum. Cerebellum.

[CIT0031] Hou P, Li Y, Zhang X, Liu C, Guan J, Li H ((2013)). Pluripotent stem cells induced from mouse somatic cells by small-molecule compounds. Science.

[CIT0032] Hu B. Y., Zhang S. C. ((2009)). Differentiation of spinal motor neurons from pluripotent human stem cells. Nat Protoc.

[CIT0033] Hunsberger J, Efthymiou A. G., Malik N, Behl M, Mead I. L., Zeng X ((2015)). Induced pluripotent stem cell models to enable in vitro models for screening in the CNS. Stem Cells Dev.

[CIT0034] Israel M. A., Yuan S. H., Bardy C, Reyna S. M., Mu Y, Herrera C ((2012)). Probing sporadic and familial Alzheimer's disease using induced pluripotent stem cells. Nature.

[CIT0035] Ito M ((1984)). The modifiable neuronal network of the cerebellum. Jpn J Physiol.

[CIT0036] Jeon I, Lee N, Li J. Y., Park I. H., Park K. S., Moon J ((2012)). Neuronal properties, in vivo effects, and pathology of a Huntington's disease patient-derived induced pluripotent stem cells. Stem Cells.

[CIT0037] Jiang J, Lv W, Ye X, Wang L, Zhang M, Yang H ((2013)). Zscan4 promotes genomic stability during reprogramming and dramatically improves the quality of iPS cells as demonstrated by tetraploid complementation. Cell Res.

[CIT0038] Kamao H, Mandai M, Okamoto S, Sakai N, Suga A, Sugita S ((2014)). Characterization of human induced pluripotent stem cell-derived retinal pigment epithelium cell sheets aiming for clinical application. Stem Cell Rep.

[CIT0039] Koch P, Breuer P, Peitz M, Jungverdorben J, Kesavan J, Poppe D ((2011)). Excitation-induced ataxin-3 aggregation in neurons from patients with Machado-Joseph disease. Nature.

[CIT0040] Koch P, Opitz T, Steinbeck J. A., Ladewig J, Brustle O ((2009)). A rosette-type, self-renewing human ES cell-derived neural stem cell with potential for in vitro instruction and synaptic integration. Proc Natl Acad Sci U S A.

[CIT0041] Kordower J. H., Chu Y, Hauser R. A., Freeman T. B., Olanow C. W. ((2008)). Lewy body-like pathology in long-term embryonic nigral transplants in Parkinson's disease. Nat Med.

[CIT0042] Kriks S, Shim J. W., Piao J, Ganat Y. M., Wakeman D. R., Xie Z ((2011)). Dopamine neurons derived from human ES cells efficiently engraft in animal models of Parkinson's disease. Nature.

[CIT0043] Ku S, Soragni E, Campau E, Thomas E. A., Altun G, Laurent L. C. ((2010)). Friedreich's ataxia induced pluripotent stem cells model intergenerational GAATTC triplet repeat instability. Cell Stem Cell.

[CIT0044] Ladewig J, Koch P, Brustle O ((2013)). Leveling Waddington: The emergence of direct programming and the loss of cell fate hierarchies. Nat Rev Mol Cell Biol.

[CIT0045] Lancaster M. A., Renner M, Martin C. A., Wenzel D, Bicknell L. S., Hurles M. E. ((2013)). Cerebral organoids model human brain development and microcephaly. Nature.

[CIT0046] Lee G, Papapetrou E. P., Kim H, Chambers S. M., Tomishima M. J., Fasano C. A. ((2009)). Modelling pathogenesis and treatment of familial dysautonomia using patient-specific iPSCs. Nature.

[CIT0047] Lee K. S., Zhou W, Scott-McKean J. J., Emmerling K. L., Cai G. Y., Krah D. L. ((2012)). Human sensory neurons derived from induced pluripotent stem cells support varicella-zoster virus infection. PLoS one.

[CIT0048] Leiner H. C. ((2010)). Solving the mystery of the human cerebellum. Neuropsychology Rev.

[CIT0049] Li X. J., Du Z. W., Zarnowska E. D., Pankratz M, Hansen L. O., Pearce R. A. ((2005)). Specification of motoneurons from human embryonic stem cells. Nat Biotechnol.

[CIT0050] Li X. J., Hu B. Y., Jones S. A., Zhang Y. S., Lavaute T, Du Z. W. ((2008)). Directed differentiation of ventral spinal progenitors and motor neurons from human embryonic stem cells by small molecules. Stem Cells.

[CIT0051] Malik N, Efthymiou A. G., Mather K, Chester N, Wang X, Nath A ((2014)). Compounds with species and cell type specific toxicity identified in a 2000 compound drug screen of neural stem cells and rat mixed cortical neurons. Neurotoxicology.

[CIT0052] Manto M ((2008)). The cerebellum, cerebellar disorders, and cerebellar research–two centuries of discoveries. Cerebellum.

[CIT0053] Marchetto M. C., Carromeu C, Acab A, Yu D, Yeo G. W., Mu Y ((2010)). A model for neural development and treatment of Rett syndrome using human induced pluripotent stem cells. Cell.

[CIT0054] Mendonca L. S., Nobrega C, Hirai H, Kaspar B. K., Pereira de Almeida L ((2015)). Transplantation of cerebellar neural stem cells improves motor coordination and neuropathology in Machado-Joseph disease mice. Brain.

[CIT0055] Moretti A, Bellin M, Welling A, Jung C. B., Lam J. T., Bott-Flugel L ((2010)). Patient-specific induced pluripotent stem-cell models for long-QT syndrome. N Engl J Med.

[CIT0056] Muguruma K, Nishiyama A, Kawakami H, Hashimoto K, Sasai Y ((2015)). Self-organization of polarized cerebellar tissue in 3D culture of human pluripotent stem cells. Cell Rep.

[CIT0057] Muguruma K, Nishiyama A, Ono Y, Miyawaki H, Mizuhara E, Hori S ((2010)). Ontogeny-recapitulating generation and tissue integration of ES cell-derived Purkinje cells. Nat Neurosci.

[CIT0058] Okita K, Matsumura Y, Sato Y, Okada A, Morizane A, Okamoto S ((2011)). A more efficient method to generate integration-free human iPS cells. Nat Methods.

[CIT0059] Patani R, Hollins A. J., Wishart T. M., Puddifoot C. A., Alvarez S, de Lera A. R. ((2011)). Retinoid-independent motor neurogenesis from human embryonic stem cells reveals a medial columnar ground state. Nature commun.

[CIT0060] Patani R, Lewis P. A., Trabzuni D, Puddifoot C. A., Wyllie D. J., Walker R ((2012)). Investigating the utility of human embryonic stem cell-derived neurons to model ageing and neurodegenerative disease using whole-genome gene expression and splicing analysis. J Neurochem.

[CIT0061] Perrier A. L., Tabar V, Barberi T, Rubio M. E., Bruses J, Topf N ((2004)). Derivation of midbrain dopamine neurons from human embryonic stem cells. Proc Natl Acad Sci U S A.

[CIT0062] Perrier A, Peschanski M ((2012)). How can human pluripotent stem cells help decipher and cure Huntington's disease?. Cell Stem Cell.

[CIT0063] Pomp O, Brokhman I, Ben-Dor I, Reubinoff B, Goldstein R. S. ((2005)). Generation of peripheral sensory and sympathetic neurons and neural crest cells from human embryonic stem cells. Stem Cells.

[CIT0064] Reubinoff B. E., Itsykson P, Turetsky T, Pera M. F., Reinhartz E, Itzik A ((2001)). Neural progenitors from human embryonic stem cells. Nat Biotechnol.

[CIT0065] Ross C. A., Akimov S. S. ((2014)). Human-induced pluripotent stem cells: Potential for neurodegenerative diseases. Hum Mol Genet.

[CIT0066] Ruigrok T. J. ((2011)). Ins and outs of cerebellar modules. Cerebellum.

[CIT0067] Salero E, Hatten M. E. ((2007)). Differentiation of ES cells into cerebellar neurons. Proc Natl Acad Sci U S A.

[CIT0068] Sanchez-Danes A, Richaud-Patin Y, Carballo-Carbajal I, Jimenez-Delgado S, Caig C, Mora S ((2012)). Disease-specific phenotypes in dopamine neurons from human iPS-based models of genetic and sporadic Parkinson's disease. EMBO Mol Med.

[CIT0069] Schlaeger T. M., Daheron L, Brickler T. R., Entwisle S, Chan K, Cianci A ((2015)). A comparison of non-integrating reprogramming methods. Nat Biotechnol.

[CIT0070] Schmahmann J. D. ((1997)). Rediscovery of an early concept. Int Rev Neurobiol.

[CIT0071] Schondorf D. C., Aureli M, McAllister F. E., Hindley C. J., Mayer F, Schmid B ((2014)). iPSC-derived neurons from GBA1-associated Parkinson's disease patients show autophagic defects and impaired calcium homeostasis. Nat Commun.

[CIT0072] Schorge S, van de Leemput J, Singleton A, Houlden H, Hardy J ((2010)). Human ataxias: A genetic dissection of inositol triphosphate receptor (ITPR1)-dependent signaling. Trends Neurosci.

[CIT0073] Schwartz R. E., Fleming H. E., Khetani S. R., Bhatia S. N. ((2014)). Pluripotent stem cell-derived hepatocyte-like cells. Biotechnol Adv.

[CIT0074] Schwartz S. D., Hubschman J. P., Heilwell G, Franco-Cardenas V, Pan C. K., Ostrick R. M. ((2012)). Embryonic stem cell trials for macular degeneration: a preliminary report. Lancet.

[CIT0075] Shi Y, Kirwan P, Livesey F. J. ((2012)). Directed differentiation of human pluripotent stem cells to cerebral cortex neurons and neural networks. Nat Protoc.

[CIT0076] Soldner F, Laganiere J, Cheng A. W., Hockemeyer D, Gao Q, Alagappan R ((2011)). Generation of isogenic pluripotent stem cells differing exclusively at two early onset Parkinson point mutations. Cell.

[CIT0077] Strata P ((2015)). The emotional cerebellum. Cerebellum.

[CIT0078] Strick P. L., Dum R. P., Fiez J. A. ((2009)). Cerebellum and nonmotor function. Annu Rev Neurosci.

[CIT0079] Su H. L., Muguruma K, Matsuo-Takasaki M, Kengaku M, Watanabe K, Sasai Y ((2006)). Generation of cerebellar neuron precursors from embryonic stem cells. Dev Biol.

[CIT0080] Tabar V, Studer L ((2014)). Pluripotent stem cells in regenerative medicine: Challenges and recent progress. Nat Rev Genet.

[CIT0081] Takahashi K, Tanabe K, Ohnuki M, Narita M, Ichisaka T, Tomoda K ((2007)). Induction of pluripotent stem cells from adult human fibroblasts by defined factors. Cell.

[CIT0082] Ten Donkelaar H. J., Lammens M ((2009)). Development of the human cerebellum and its disorders. Clin Perinatol.

[CIT0083] Teng S, Liu C, Krettek C, Jagodzinski M ((2014)). The application of induced pluripotent stem cells for bone regeneration: Current progress and prospects. Tissue Eng Part B Rev.

[CIT0084] Timmann D ((2012)). [Contribution of the cerebellum to cognition]. Fortschr Neurol Psychiatr.

[CIT0085] Valensi-Kurtz M, Lefler S, Cohen M. A., Aharonowiz M, Cohen-Kupiec R, Sheinin A ((2010)). Enriched population of PNS neurons derived from human embryonic stem cells as a platform for studying peripheral neuropathies. PLoS One.

[CIT0086] Vierbuchen T., Wernig M ((2012)). Molecular roadblocks for cellular reprogramming. Mol Cell.

[CIT0087] Voogd J ((2014)). What we do not know about cerebellar systems neuroscience. Front Syst Neurosci.

[CIT0088] Voogd J, Gerrits N. M., Ruigrok T. J. ((1996)). Organization of the vestibulocerebellum. Ann N Y Acad Sci.

[CIT0089] Wang S, Wang B, Pan N, Fu L, Wang C, Song G ((2015)). Differentiation of human induced pluripotent stem cells to mature functional Purkinje neurons. Sci Rep.

[CIT0090] Warren L, Manos P. D., Ahfeldt T, Loh Y. H., Li H, Lau F ((2010)). Highly efficient reprogramming to pluripotency and directed differentiation of human cells with synthetic modified mRNA. Cell Stem Cell.

[CIT0091] White J. J., Sillitoe R. V. ((2013)). Development of the cerebellum: from gene expression patterns to circuit maps. Wiley Interdiscip Rev Dev Biol.

[CIT0092] Woodard C. M., Campos B. A., Kuo S. H., Nirenberg M. J., Nestor M. W., Zimmer M ((2014)). iPSC-derived dopamine neurons reveal differences between monozygotic twins discordant for Parkinson's disease. Cell Rep.

[CIT0093] Wurst W, Bally-Cuif L ((2001)). Neural plate patterning: Upstream and downstream of the isthmic organizer. Nat Rev Neurosci.

[CIT0094] Xie Y. Z., Zhang R. X. ((2015)). Neurodegenerative diseases in a dish: The promise of iPSC technology in disease modeling and therapeutic discovery. Neurol Sci.

[CIT0095] Yamanaka S, Blau H. M. ((2010)). Nuclear reprogramming to a pluripotent state by three approaches. Nature.

[CIT0096] Yan Y, Yang D, Zarnowska E. D., Du Z, Werbel B, Valliere C ((2005)). Directed differentiation of dopaminergic neuronal subtypes from human embryonic stem cells. Stem Cells.

[CIT0097] Yang Y. M., Gupta S. K., Kim K. J., Powers B. E., Cerqueira A, Wainger B. J. ((2013)). A small molecule screen in stem-cell-derived motor neurons identifies a kinase inhibitor as a candidate therapeutic for ALS. Cell Stem Cell.

[CIT0098] Yu J, Hu K, Smuga-Otto K, Tian S, Stewart R, Slukvin I. I., Thomson J. A. ((2009)). Human induced pluripotent stem cells free of vector and transgene sequences. Science.

[CIT0099] Zeng X, Hunsberger J. G., Simeonov A, Malik N, Pei Y, Rao M ((2014)). Concise review: modeling central nervous system diseases using induced pluripotent stem cells. Stem Cells Transl Med.

[CIT0100] Zhang S. C., Wernig M, Duncan I. D., Brustle O, Thomson J. A. ((2001)). In vitro differentiation of transplantable neural precursors from human embryonic stem cells. Nat Biotechnol.

